# IL‐33/ST2 receptor‐dependent signaling in the development of pulmonary hypertension in Sugen/hypoxia mice

**DOI:** 10.14814/phy2.15185

**Published:** 2022-02-12

**Authors:** Cynthia S. Indralingam, Alma K. Gutierrez‐Gonzalez, Scott C. Johns, Tzuhan Tsui, Daniel T. Cannon, Mark M. Fuster, Timothy D. Bigby, Patricia A. Jennings, Ellen C. Breen

**Affiliations:** ^1^ Medicine University of California, San Diego La Jolla California USA; ^2^ VA San Diego Healthcare System La Jolla California USA; ^3^ School of Exercise and Nutritional Sciences San Diego State University San Diego California USA; ^4^ Division of Pulmonary & Critical Care University of California, San Diego La Jolla California USA; ^5^ Chemistry and Biochemistry University of California, San Diego La Jolla California USA

**Keywords:** endothelial cells, interleukin‐33 receptor, pulmonary hypertension

## Abstract

Pulmonary arterial hypertension (PAH) is associated with significant morbidity and mortality. PAH is characterized by pulmonary artery remodeling, elevated right ventricular pressure (RVP) and, ultimately, cardiac failure. Pulmonary endothelial cells can sense danger or damage caused by mechanical injury or pathogens through alarmin cytokines. These cytokines can signal proliferation to restore barrier integrity or aberrant hyperproliferation and remodeling. We hypothesized that IL‐33 signals pulmonary artery endothelial cells to proliferate under hypertensive conditions during the remodeling response and rise in RVP. To test this hypothesis, pulmonary hypertension (PH) was induced in C57Bl/6J, IL‐33 receptor gene deleted (ST2^−/−^) and MYD88 gene deleted (MYD88^−/−^) mice by exposure to 10% O_2_ and SU5416 injections (SUHX). RVP, arterial wall thickness, endothelial cell proliferation and IL‐33 levels and signaling were evaluated. In response to SUHX. RVP increased in C57Bl/6J mice in response to SUHX (49% male and 70% female; *p* < 0.0001) and this SUHX response was attenuated in ST2^−/−^ mice (29% male *p* = 0.003; 30% female *p* = 0.001) and absent in MYD88^−/−^ mice. Wall thickness was increased in SUHX C57Bl/6J mice (*p* = 0.005), but not in ST2^−/−^ or MYD88^−/−^ mice. Proliferating cells were detected in C57Bl/6J mice by flow cytometry (CD31^+^/BrDU^+^; *p* = 0.02) and immunofluorescence methods (Ki‐67+). IL‐33 was increased by SUHX (*p* = 0.03) but a genotype effect was not observed (*p* = 0.76). We observed that in hPAECs, IL‐33 expression is regulated by both IL‐33 and DLL4. These data suggest IL‐33/ST2 signaling is essential for the endothelial cell proliferative response in PH.

## Graphical abstract

The IL‐33/ST2 pathway is essential for the early endothelial cell proliferative response in Sugen/hypoxia‐induced pulmonary hypertension that leads to remodeling of the small resistance pulmonary arteries. The intracellular toll‐like receptor adaptor protein, MYD88, synergizes with the IL‐33/ST2 receptor complex to regulate right ventricle pressure and right ventricle contractility and remodeling.

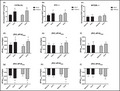

## 1 INTRODUCTION

The classifications of Pulmonary hypertension (PH), most recently reviewed and revised at the 2018 World Symposium on Pulmonary Hypertensio, assist in the diagnosis and management of this disease (Galiè et al., 2019). Pulmonary arterial hypertension (PAH), including idiopathic and heritable PAH, falls into the Group 1, which defines the PH as a result of increased pulmonary vascular resistance (PVR; Ryan et al., 2012; Simonneau et al., 2019). This is a disease initiated by aberrant pulmonary arterial wall remodeling that leads to high blood pressures in the lungs and right heart (Lai et al., 2014). PAH can develop in both males and females of any age but is more prevalent among females (Lai et al., 2014). Some common treatment options for Group 1 patients include pulmonary artery vasodilators, such as phosphodiesterase‐5 inhibitors, endothelin receptor antagonists, and prostacyclin analogs, all of which open narrowed blood vessels to allow ease of blood flow and ultimately lower pulmonary blood pressure (Rose‐Jones & Mclaughlin, 2015). However, PAH is still an oftentimes fatal disease with no effective cure (Raja & Raja, 2011). In recent years, researchers have focused on understanding the inflammation and impaired immunity that may underlie PAH in an effort to develop new therapeutics (Rabinovitch et al., 2014).

Alarmins are a group of cytokines expressed in endothelial and epithelial cell barriers, which are the protective barriers of the lung, and are part of the first line defense against respiratory infections and injury (Saïd‐Sadier & Ojcius, 2012; Yang et al., 2017). One theory is that acute localized inflammatory events may be initiating the disease progression factors in the pathologic events leading to severe PAH (Tamosiuniene et al., 2011). This is supported by the reports of elevated levels of alarmins in the serum of PAH patients and animal models. These alarmins include interleukin‐1β (IL‐1β), high‐mobility group box 1, S100A8/9 and interleukin‐33 (IL‐33) and, all are proposed to play a significant role in cellular mechanisms leading to PAH (Bauer et al., 2013; Humbert et al., 1995; Liu et al., 2018; Nakamura et al., 2018; Titone et al., 2014). However, the steps leading from tissue damage to alarmin activation and ultimately to a damaging hyperproliferative response are unknown. Of particular note, the cytokine IL‐33 appears to have a critical role in other lung diseases including cigarette smoke‐induced chronic obstructive pulmonary disease and respiratory infections (Cohen et al., 2015; Kearley et al., 2015; Lee et al., 2019). IL‐33 was recently discovered as a cytokine that signals through the ST2 receptor to maintain a balance of the pro‐ and anti‐inflammatory activities and, IL‐33 is critical to pulmonary homeostasis by promoting both an immunological response and governing tissue repair (Molofsky et al., 2015; Schmitz et al., 2005).

IL‐33 is a ligand of the ST2 receptor that activates immune responses in many cells including endothelial cells (Kakkar & Lee, 2008). ST2 forms a heterodimer along with the IL‐1 receptor accessory protein (Chackerian et al., 2007) leading to the dimerization of the toll‐like receptor (TIR) domain (Griesenauer & Paczesny, 2017). IL‐33/ST2 signal transduction results in the recruitment of adaptor proteins, including the myeloid differentiation factor 88 (MyD88), to regulate downstream kinases eventually stimulating the activation of NF‐kB to regulate the expression of a subset of cytokines, including IL‐33 (Griesenauer & Paczesny, 2017; Kakkar & Lee, 2008). However, there are more than one signaling pathways to increase the expression of IL‐33 and, Notch3 signaling has been implicated in smooth muscle cell proliferation in PAH (Li et al., 2009). Notch signaling is a known driver of the expression of IL‐33 and DLL4. The latter is a Nnotch ligand expressed in endothelial cells and shown to be a strong inducer of IL‐33 in endothelial cells lines (Smith et al., 2015).

Despite observations of increased IL‐33 levels with PAH (11, 12), the role of IL‐33 in regulating the endothelial cell proliferative response in vivo has not been established. In this study, we hypothesized that IL‐33 is essential for initiating endothelial cell proliferation in PH that contributes to small resistance artery remodeling in the lungs. SU5416/hypoxia conditions (SUHX) were used to model PH in wildtype (WT), IL‐33 receptor ST2 gene deleted (ST2^−/−^) or its partner MYD88 gene deleted (MYD88^−/−^) mice. Lung endothelial cell proliferation, arterial wall thickening, and mature IL‐33 levels were measured. The potential autocrine role of IL‐33 in human pulmonary artery endothelial cell (hPAEC) was tested in vitro. We present data in support of IL‐33/ST2‐dependent endothelial cell proliferation in PH.

## 2 METHODS

### 2.1 Animals

Animal protocols were approved by the VASDHS IACUC. Adult, age 2–4 months, WT C57BL/6J from Jackson Laboratory, ST2^−/−^ (Hoshino et al., 1999), and MYD88^−/−^ (Kawai et al., 1999) male and female mice were studied. Mice were housed 4–5 animals/cage in ambient temperatures 24°C, with 12 h of light per day and water and standard chow ad libitum.

### 2.2 Hypoxia/Sugen 5416 (SUHX) mice

Wildtype and mutant mice were treated weekly, three times with Sugen5416 (20 mg/kg in DMSO, *s*.*c*., Cayman Chemical) and normobaric, 10% O_2_. Mice were weighed at the end of the exposure period (Figure S1). Right ventricle pressures were measured as previously described (Breen et al., 2017). The right ventricular chamber was accessed by way of Sastry et al. (2006) with a small pressure‐conductance transducer. Briefly, mice were anesthetized and an incision was made on the midline of the neck to allow the right jugular vein to be exposed by blunt dissection. A distal tie (6–0 silk suture) on the vein was made to serve as a retractor while a loose silk knot was placed at the proximal end that was used to occlude the vein temporally. A small incision was made between the two sutures and a 1.4F pressure‐conductance transducer (Millar SPR‐839) was inserted and advanced into the vessel and to the RV cavity. The catheter was secured by the proximal suture once the RV pressure waveform was identified. Right ventricular pressures (RVPs) were recorded, converted digitally via an analog‐digital converter (emka Technologies, IOX 1.8), and stored on a computer for analyses. Upon completion of data collection, the mouse was euthanized by an overdose of isoflurane (5%).

### 2.3 Lung histology and small artery wall thickness

Right lungs were perfused with PBS and airway‐fixed with 0.75 ml buffered aqueous zinc formalin (Z‐fix, Anatech Ltd). Paraffin‐embedded (5 μm) sections were stained with hematoxylin and eosin (H&E; Thermo Fisher Scientific) and scanned with a Hamamatsu 2.0‐HT Nanozoomer Slide Scanning System. The total vascular area at the adventitial border and the lumen area at the basement membrane were outlined and measured using the Nanozoomer Digital Pathology NDP.view2 software. The wall thickness in arteries (<50 μm diameter) = (total vascular area − lumen area)/total vascular area (Ma et al., 2011).

### 2.4 Proliferating cells

Paraffin‐embedded lung serial sections were immunostained with Ki67 and α‐smooth muscle actin (α‐SMA; Supplemental Data) antibodies. The endothelial cell mitotic index was measured by flow cytometer analysis. Briefly, mice were injected with 5‐bromo‐2′deoxyuridine (BrdU) for seven consecutive days (50 mg/kg, i.p. Sigma‐Aldrich). Left pulmonary lobes were digested with 0.2% Collagenase I (Sigma‐Aldrich) and 100 ug/ml DNase type I (Sigma‐Aldrich) in DMEM (GIBCO) at 37°C for 30–45 min, passed over a 40 μm cell strainer (VMR International), washed and centrifuged. Cells were incubated with the following antibodies: anti‐CD31 (dilution 1:100, Cat. No. 550274; BD Pharmingen), CD31‐FITC (dilution 1:100, clone 390, 11‐0311‐82; eBioscience) and anti‐BrdU‐PE (dilution 1:100, clone: BU20A; eBioscience), detected with a Cytoflex or Guava easyCyte 8HT (Millipore) and analyzed using FlowJo Software (Tri Star Inc.).

### 2.5 Western blot analysis

Left lung lobe extracts were prepared in RIPA Buffer with Protease Inhibitor (Sigma‐Aldrich), electrophoresed by SDS‐PAGE with Tris Carboxy Ethyl Phosphene (TCEP; Thermo Fisher Scientific), electrotransferred to a Fluorescence PVDF membrane (MilliporeSigma), blocked for 60 min (Blocking Buffer; LI‐COR Biosciences) and probed with the following antibodies: IL‐33 (mouse mAb, 1:200 dilution, Nessy‐1 ALX‐804‐840/1; Enzo Life Sciences, Inc.), ST2 (goat pAb, 1:1000, Human ST2/IL‐33R AF523; R&D Systems), MYD88 (goat pAb, 1:1000, Mouse/Rat MyD88 AF3109; R&D Systems) and *α*‐tubulin (rabbit Ab, 1:2000 dilution; Cell Signaling Technology) overnight at 4°C. Signals were detected with IRDye800‐conjugated secondary antibodies (LI‐COR Biosciences) and visualized using the LI‐COR Odyssey imaging system (LI‐COR Biosciences). Signals were quantified using Image J and normalized to *α*‐tubulin.

### 2.6 hPAEC culture experiments

hPAEC cell experiments are described in the Supplemental Data.

### 2.7 Statistical analysis

**FIGURE 1:**
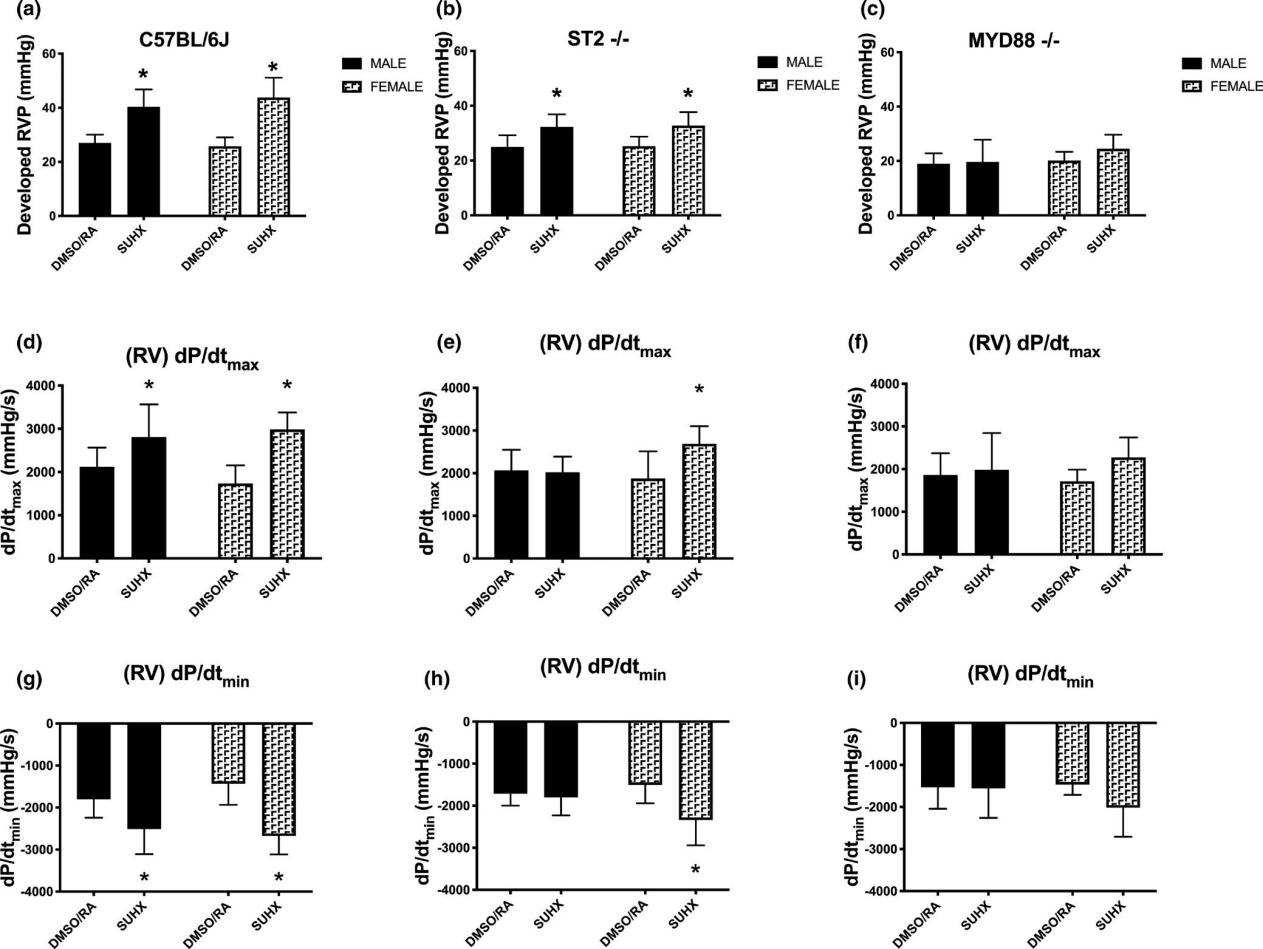
Pulmonary hypertension parameters in SUHX mice. Developed right ventricular pressures in (a) C57Bl/6J, (b) ST2^−/−^, and (c) MYD88^−/−^ mice in response to SUHX and DMSO/RA. Right ventricle contractility [(RV) + dP/dt] in response to SUHX and DMSO/RA treatments in (d) C57Bl/6J, (e) ST2^−/−^, and (f) MYD88^−/−^ mice. Right ventricle relaxation [(RV) − dP/dt] in response to SUHX and DMSO/RA in (g) C57Bl/6J, (h) ST2^−/−^, and (i) MYD88^−/−^ mice. Values are presented as mean ± *SD* (*n* = 15 male, *n* = 8 female DMSO/RA; *n* = 14 male, *n* = 6 female SUHX). *Significant difference between SUHX and DMSO/RA within a sex (*p* < 0.05)

**FIGURE 2:**
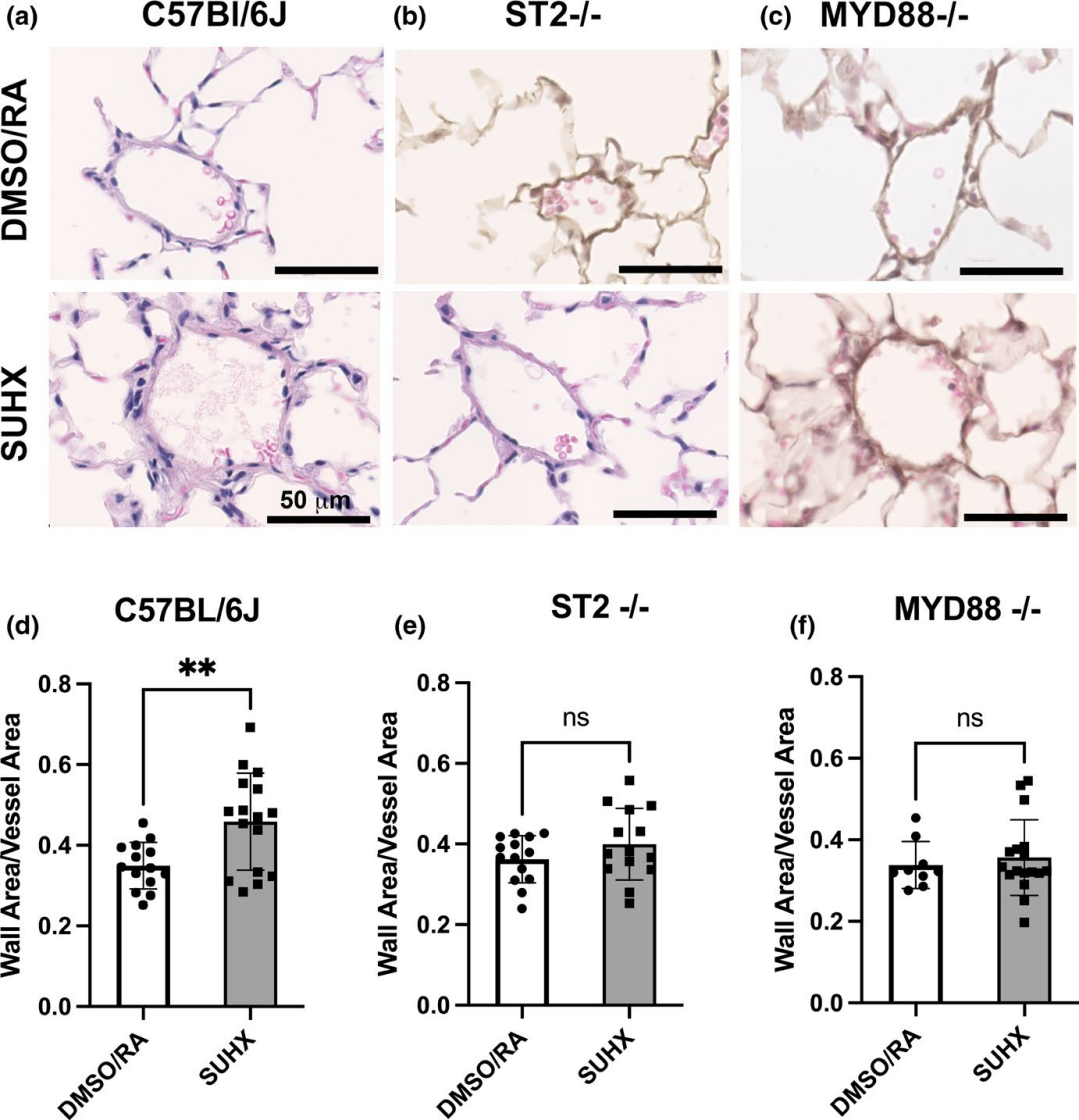
Pulmonary vascular remodeling‐wall thickness in the small arteries (<50 μm in diameter) is shown in lung sections stained with hematoxylin and eosin (a) C57Bl/6J, (b) ST2^−/−^ and (c) MYD88^−/−^ mice under DMSO/RA and SUHX treatments. Quantitation of wall thickness in (d) C57Bl/6J, (e) ST2^−/−^ and (f) MYD88^−/−^ under DMSO/RA and SUHX conditions. Values are presented as mean ± *SD* (*n* = 9–14 DMSO; *n* = 14–17 SUHX). **Statistical significance between DMSO/RA and SUHX treatments within each genotype, *p* < 0.05

**FIGURE 3:**
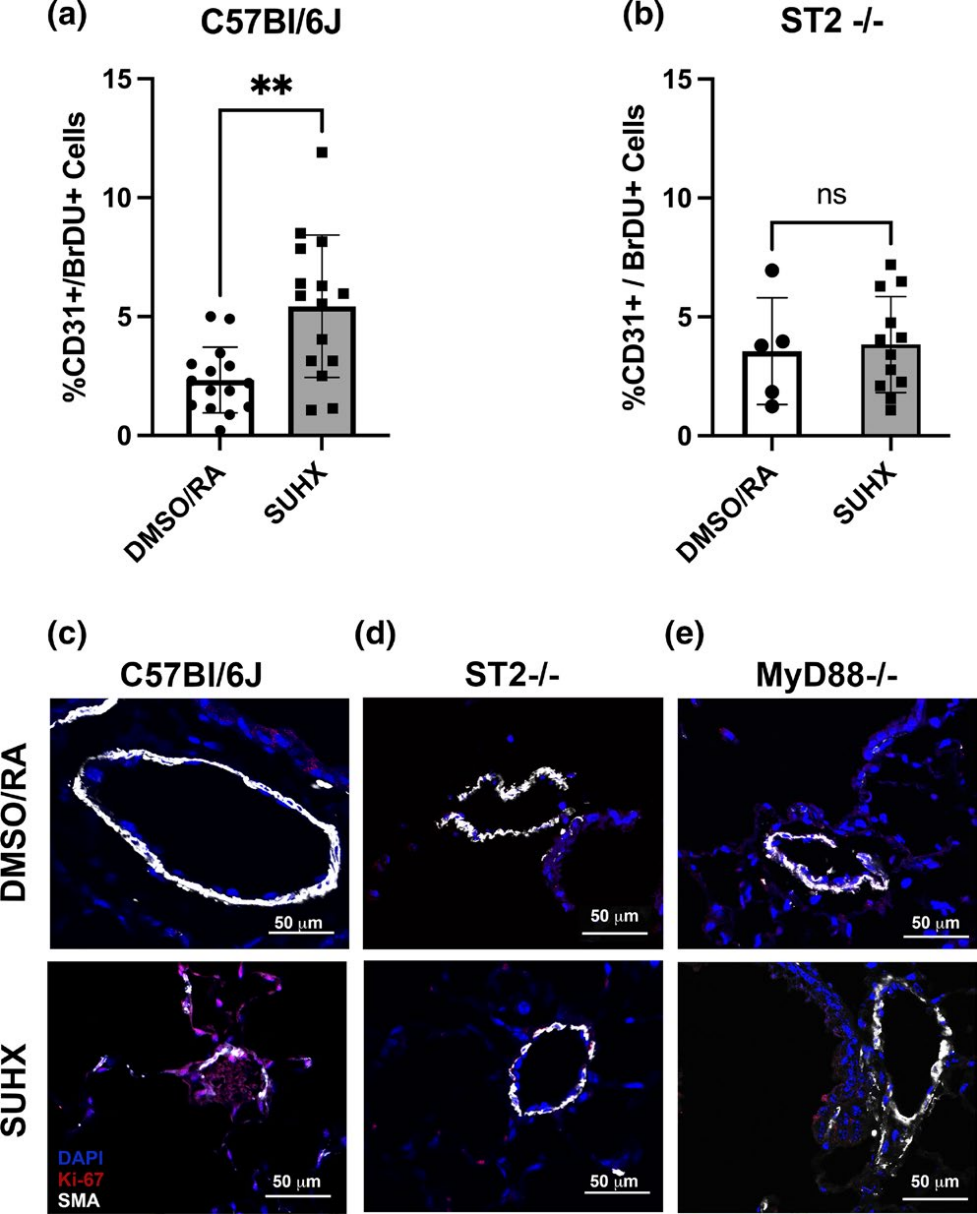
Vascular remodeling in small arteries and arterioles. Endothelial cell proliferation was evaluated by immunohistochemical and flow cytometry analysis of proliferating CD31^+^ cells. The percentage of proliferating endothelial cells (% CD31^+^/BrDU^+^) in (a) C57Bl/6J and (b) ST2^−/−^ mice under DMSO/RA and SUHX conditions. Localization of proliferating (Ki‐67) cells detected in SMA positive small arteries of (c) C57Bl/6J, (d) ST2^−/−^, and (e) MYD88^−/−^ mice under DMSO/RA and SUHX conditions. Scale bars represent 50 μm. Values are presented as the means ± *SD* (*n* = 5–15 DMSO/RA, *n* = 12–17 SUHX). **Statistical significance between DMSO/RA and SUHX treatments, *p* < 0.01

**FIGURE 4:**
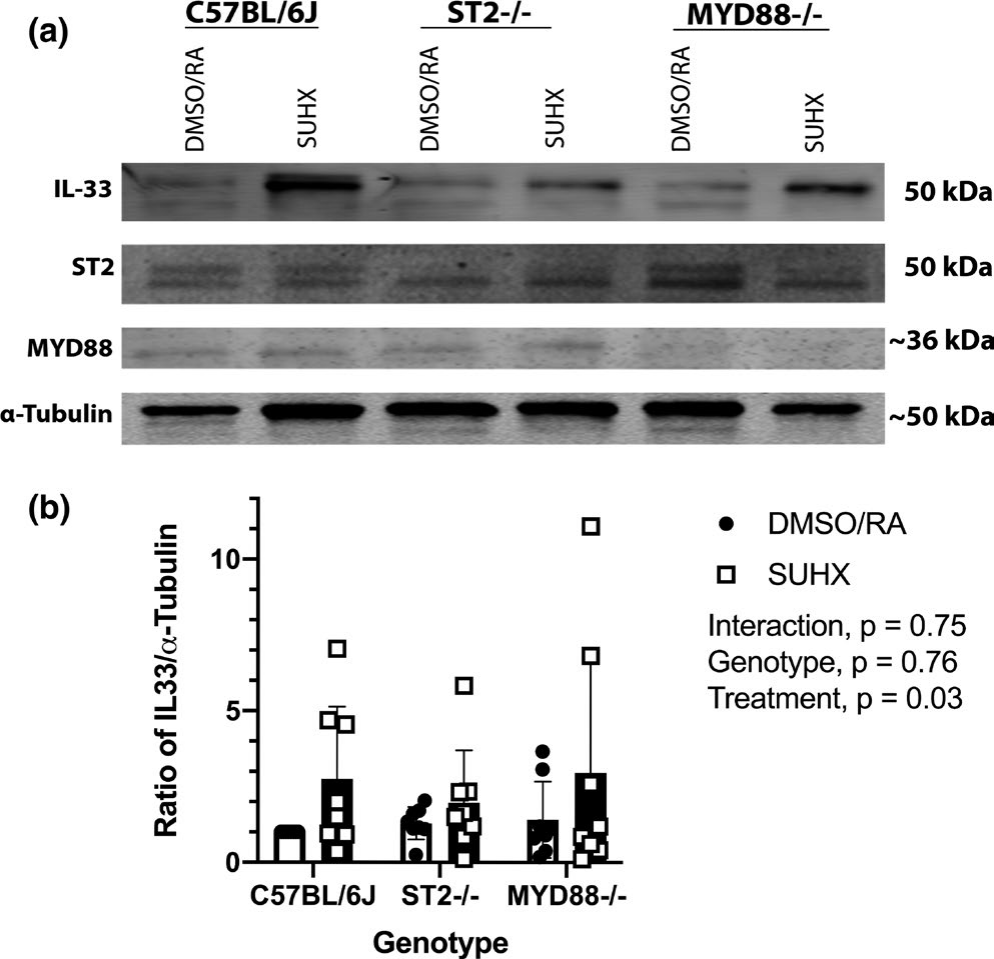
IL‐33 protein levels in whole lungs of mice exposed to SUHX (a) Western Blot representation of IL‐33, ST2, and MYD88 protein in lungs of C57BL/6J, ST2^−/−^, and MYD88^−/−^ mice exposed to either SUHX or DMSO/RA conditions (all *n* = 8). Equal loading of protein was confirmed using α‐Tubulin. (b) Quantitative analysis of IL‐33 in whole lungs. Values are presented as the means ± *SD*

**FIGURE 5:**
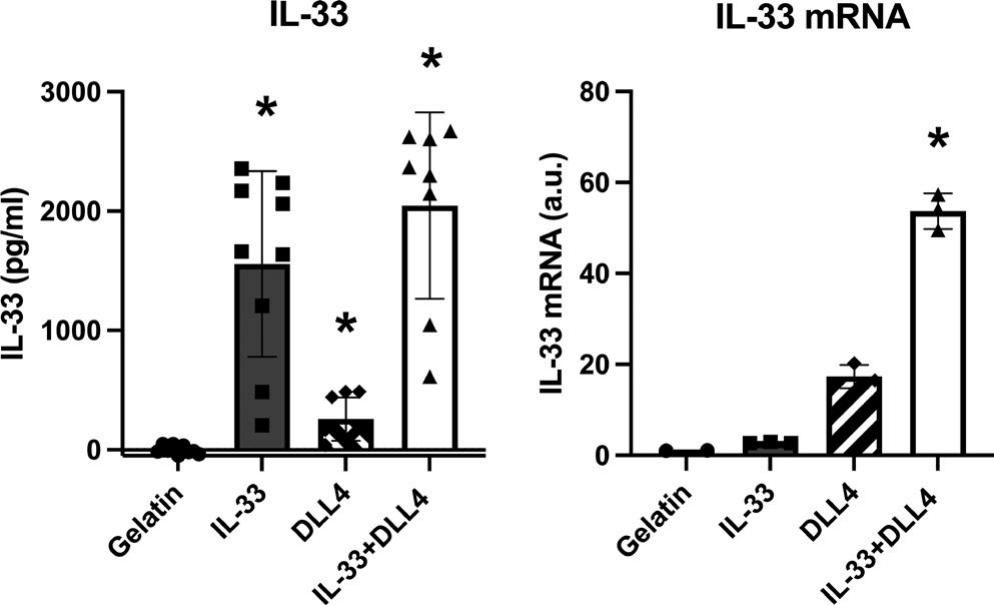
Endothelial IL‐33 autocrine expression in the presence of Notch ligand (DLL4). IL‐33 message (a) and protein levels (b) in human pulmonary artery endothelial cells after a 48 h response to exogenous IL‐33, culture on a gelatin matrix or gelatin/DLL4 matrix. Experiments were performed in triplicates with each triplicate repeated three times. Values are presented as the means ± *SD*. *Statistical significance between gelatin and IL‐33 and/or DLL4 treatments, *p* < 0.05

Data were collected by investigators that were blinded to the experimental conditions. All data are expressed as the mean ± *SD*. Two‐way (treatment, sex) analysis of variance (ANOVA) and Sidak's post‐hoc tests were used to detect differences between the RVPs (Figure 1). *T*‐tests were used to compare conditions within each genotype (Figures 2 and 3). Two‐way (genotype, treatment) ANOVA was used to analyze the differences between the IL‐33 levels in the lungs (Figure 4). Dunnett's tests were used to compare hPAEC conditions to the control (Figure 5). Statistical significance was defined as *p* < 0.05.

## 3 RESULTS

### 3.1 Increased RVP under SUHX conditions are dependent on ST2 and MYD88

Figure 1a shows that WT (C57Bl/6J) mice under SUHX conditions developed increases in RVP by 49% in males and 70% in females as compared to DMSO/RA (*p* < 0.0001). In ST2^−/−^ mice, males had a 29% increase while females had a 30% increase in developed RVP after SUHX treatments (*p* < 0.0001; Figure 1b). In MYD88^−/−^ mice, RVP did not increase above DMSO/RA values in response to 3 weeks of SUHX in both male and female mice (*p* = 0.20; Figure 1c). RV contractility (RV + dP/dt) in both male and female C57Bl/6J mice were higher with SUHX exposure than that observed with DMSO/RA treatment (*p* < 0.0001; Figure 1d). Chronic SUHX exposure also increased the RV contractility in the female ST2^−/−^ mice (*p* = 0.002) but not in the ST2^−/−^ male mice (*p* = 0.98; Figure 1e). RV contractility in both sexes of MYD88^−/−^ mice did not increase above DMSO/RA levels under SUHX conditions (male *p* = 0.86, female *p* = 0.17; Figure 1f). Similarly, only the C57Bl/6J mice, both male and female, exhibited a decrease in RV relaxation (RV − dP/dt) below DMSO/RA levels after SUHX treatment (*p* < 0.0001; Figure 1g). There were no significant changes in RV relaxation (RV − dP/dt) in the male ST2^−/−^ (*p* = 0.90) and either sex of MYD88^−/−^ mice (male *p* = 1.00, female *p* = 0.17) after SUHX exposure (Figure 1h,i). RV relaxation (RV − dP/dt) in the female ST2^−/−^ mice is decreased after SUHX treatment (*p* = 0.001). These data support the possibility that the ST2 and MYD88 gene deletion results in a protective effect against SUHX treatment.

### 3.2 Pulmonary vascular remodeling observed in C57Bl/6J mice, but not ST2^−/−^ or MYD88^−/−^ mice, under SUHX condition

Small pulmonary arteries and arterioles revealed thicker vascular walls in C57BL/6J mouse lungs in the SUHX group than C57BL/6J mice in the DMSO/RA group (*p* = 0.01; Figure 2d). These changes in wall thickness were attenuated in ST2^−/−^ and the MYD88^−/−^ arteries which did not show a difference in vascular remodeling in response to SUHX (Figure 2e,f; ST2^−/−^, DMSO/RA vs. SUHX, *p* = 0.20 and MYD88^−/−^, DMSO/RA vs. SUHX, *p* = 0.60, respectively). Figure 2a–c shows representative images of H&E stained lung sections from the mice in each group.

### 3.3 ST2 dependent endothelial cell hyperproliferation in response to SUHX

Endothelial cell ST2 dependent proliferation was observed in SUHX treated WT mice but not in DMSO/RA treated WT mice. An increase in proliferating endothelial cells (CD31^+^/BrDU^+^) is observed only in WT C57Bl/6J mice exposed to SUHX (Figure 3a, *p* = 0.001). Representative images of lung sections with immunohistochemical staining for α‐SMA and Ki‐67, a proliferation marker, show the localization of proliferating (Ki‐67+) cells located proximally to the SMA positive small arteries of C57Bl/6J but not ST2^−/−^ and MYD88^−/−^ mice under SUHX conditions (Figure 3c–e).

### 3.4 Increased IL‐33 protein in mice exposed to SUHX

IL‐33 protein was detected in lung tissue of C57BL/6J, ST2^−/−^, and MYD88^−/−^ mice exposed to either SUHX or DMSO/RA treatments. Western blots showed that IL‐33 levels were elevated in C57BL/6J mice following exposure to 3 weeks of SUHX (Figure 4a,b). Ablation of ST2 and MYD88 genes did not affect the observed increase in IL‐33 when treated with SUHX (Figure 4a,b; Treatment, *p* = 0.03). The western blot image shows a visible decrease in ST2 and MYD88 proteins confirming the knockdown of ST2 and MYD88 genes as compared to C57BL/6J (Figure 4a).

### 3.5 Ligand dependent IL‐33 autocrine regulation in hPAECs

In order to determine the role of exogenous IL‐33 and the notch ligand (DLL4) in hPAECs in IL‐33 signaling, we measured the expression of cellular IL −33 mRNA and protein in hPAECs cultured on either a gelatin or gelatin/DLL4 (1 μg/ml) matrix and stimulated with or without exogenous IL‐33 (100 ng/ml). In culture on a gelatin matrix, low levels of IL‐33 mRNA and protein are detected (Figure 5a,b). Exogenous IL‐33 added to hPAECS cultured on gelatin alone increased IL‐33 mRNA (*p* = 0.05). Culture on a DLL4‐gelatin matrix also resulted in a trend to increase in IL‐33 mRNA above culture on gelatin alone (*p* = 0.13, Figure 5a). Together exogenous IL‐33 and extracellular DLL4 treatment increase IL‐33 transcripts by 51‐fold above culture on gelatin (*p* = 0.04, Figure 5a). In Figure 5b, exogenous IL‐33 treatment led to a 1558‐fold increase in IL‐33 cell protein levels in hPAEC cells cultured on gelatin (*p* = 0.0007). Culture on a DLL4‐gelatin matrix led to a 258‐fold increased production of IL‐33 relative to that observed for culture on gelatin alone (*p* = 0.004; Figure 5b). Together, the combination of IL‐33 and DLL4 treatments signaled a 2047‐fold increase in IL‐33 protein above cells cultured on gelatin (*p* = 0.0003, Figure 5b).

## 4 DISCUSSION

While alarmins, including IL‐33, are implicated in the progression of PAH (Liu et al., 2018; Titone et al., 2014), the functional role of the IL‐33‐ST2 signaling axis in the proliferative response and subsequent remodeling have not been elucidated. Our data indicate that under chronic SUHX‐induced PH conditions in mice, the IL‐33/ST2 signaling pathway regulates a proliferative response in PAECs. This proliferative response contributes to the observed vascular wall remodeling and increased RVP that develop after 3 weeks under SUHX conditions. Inhibiting ST2 receptor expression completely prevented the SUHX‐induced proliferation and wall remodeling responses but elevated RVPs were only partially attenuated. Inhibition of the adaptor protein, MYD88, which interacts with the IL‐33/ST2 complex, completely prevented the proliferation, remodeling, and pressure responses in the SUHX model. Mature, processed IL‐33 increased in response to SUHX conditions across all genotypes. In vitro data using hPAECs suggests autocrine IL‐33 upregulation can occur through PAH associated endothelial DLL4 notch signaling.

### 4.1 IL‐33 regulates the endothelial cell proliferative response in SUHX‐induced PH

Our experiments show that IL‐33 is essential for the proliferative response in PH induced by SUHX. There is a hyperproliferative response in the development of PH (Heath & Edwards, 1958; Perros et al., 2019; Tuder et al., 1994). Endothelial cells isolated from idiopathic PAH patients have a greater proliferative rate than observed for healthy patients that is linked to increased cell survival and an amplified cell cycle progression (Masri et al., 2007). IL‐33 is released extracellularly after endothelial cell damage due to injury or mechanical strain (Cayrol & Girard, 2009). Thus, our studies suggest that damage to the endothelium and subsequent increased tension as the pulmonary artery remodel leads to a persistent proliferative response which is dependent on IL‐33/ST2 signaling. This is supported by our finding that ST2 gene deficient mice are protected from the endothelial cell proliferative response that occurs in the lungs of SUHX exposed WT mice (Figure 3). This observation complements existing data collected in mice deficient in MYD88, an adaptor protein upstream in the IL‐33 signaling pathway, which shows that following exposure to chronic hypoxia that there are fewer proliferating cells in the pulmonary vessels in Myd88 null mice than that observed for WT mice (Parpaleix et al., 2016). Treatment with exogenous IL‐33 has also been shown to directly increase human pulmonary artery endothelial cell (hPAEC) proliferation cultured under normoxic conditions (Liu et al., 2018), and gene deletion of IL‐33 in cultured hPAECs attenuates proliferation under both normoxia and hypoxia conditions (Liu et al., 2018). Taken together, these in vitro studies support an IL‐33 regulated mechanism in the proliferation of endothelial cells in PH. We now show the importance of IL‐33/ST2 signaling in regulating this key proliferative response in the SUHX‐induce mouse model of PH.

### 4.2 Arterial wall remodeling is dependent on IL‐33

Acute inflammation associated with increased TNFα and IL‐6 levels has been suggested to be a key factor for the development of PAH (Tamosiuniene et al., 2011). Our experiments suggest that PH vascular remodeling is dependent on the alarmin IL‐33 located in the pulmonary endothelium (Martin & Martin, 2016). We show that ablation of ST2 or MYD88 prevented an increase in the overall thickness of the resistance arteries (<50 μm) after inducing PH in our model (SUHX; Figure 3). This suggests that IL‐33 contributes to the pathogenesis of PH as in the absence of the ST2 receptor arterial wall remodeling was completely prevented. Similarly, Liu and colleagues observed that pulmonary vascular remodeling in PH induced by hypoxia alone is attenuated in ST2 null mice (Liu et al., 2018). Our study is focused on the endothelial proliferative response that could initiate vascular remodeling through the activation of IL‐33 and potentially through additional alarmins such as HMGB‐1, IL‐1β, and S100A8/A9 (Bauer et al., 2013; Humbert et al., 1995; Nakamura et al., 2018).

### 4.3 Right ventricular function is dependent on the IL‐33/ST2 signaling axis

A persistent increase in the afterload on the right heart activates mechanisms that results in RV hypertrophy that over time transition to RV dilation (Chin et al., 2005; Klinger & Hill, 1991). These vascular changes force the right heart to increase its rate of contractility, and once these contractile reserves are exhausted, an irreversible decrease in cardiac function develops (Bogaard et al., 2009; Chin et al., 2005; Guyton et al., 1954; Lee et al., 2019). Thus, by monitoring the instantaneous rates of contraction and relaxation we can learn more about the progression of PH. Our observations show equivalent elevations in developed RVP in both male and female C57Bl/6J SUHX mice (Figure 1). The developed RVP response observed in ST2^−/−^ mice was only half the level observed in WT mice when exposed to SUHX (Figure 1). Ablation of MYD88, an upstream adaptor protein, completely prevented developed RVP increases in this PH model (Figure 1). The elevated RVP in response to chronic hypoxia alone without Sugen5416 also depends on ST2 and MYD88 (Liu et al., 2018; Parpaleix et al., 2016). Our findings show that developed RVP in the SUHX mouse model is only partially dependent on ST2. Further, evaluation of the dynamic contractility suggests that elevated RVPs are accompanied by changes in RV contraction (d*P*/d*t*_min_) and RV relaxation (d*P*/d*t*_max_) and, these changes are equivalent in both the male and female C57Bl/6J mice. Interestingly, the rate of RV contraction (d*P*/d*t*_min_) is blunted in the male but not the female mice that are deficient in the ST2 receptor. Female mice also demonstrate protection from the weight loss seen in male SUHX mice (Figure S1). Both sexes of the MYD88^−/−^ mice did not demonstrate an increase in the RV contraction (d*P*/d*t*_min_) and RV relaxation (d*P*/d*t*_max_) after SUHX treatments (Figure 1). Ex vivo studies in hearts isolated from SUHX treated rats also show increases in developed right ventricular systolic pressure (RVSP) and d*P*/d*t*_max_ (Neto‐Neves et al., 2017). Our in vivo data indicate that 3 weeks of SUHX treatments results in not only equivalent increases in RVP in both male and female mice but also sex differences in RV contraction (d*P*/d*t*_min_) that are regulated by the IL‐33/ST2 pathway.

### 4.4 IL‐33 is released under SUHX conditions

The full‐length form of IL‐33 is 10‐ to 30‐fold less potent than the processed form (18–21 kDA; Liew et al., 2010). Western blots of the lung tissue of WT mice under PH conditions (SUHX) show an increase in the processed form of IL‐33 (Figure 4). Interestingly, the western blot of lung tissues from ST2^−/−^ and MYD88^−/−^ mice in our experiment also show that IL‐33 levels are increased by SUHX conditions (Figure 4). This is interesting because our data show that the PH condition of SUHX does indeed cause IL‐33 signaling to be upregulated (Figure 4) and IL‐33 is upregulated in PH patients (Liu et al., 2018; Titone et al., 2014). However, the upregulation of IL‐33 in SUHX exposed ST2^−/−^ and MYD88^−/−^ mice did not result in a proliferative response or vascular remodeling (Figures 2 and 3). Thus, our data suggest that IL‐33/ST2 signaling is essential for the endothelial proliferative response but, additional IL‐33 signaling pathways or signals initiated by additional alarmins or growth factors may contribute to the later progression of the remodeling response in the small pulmonary arteries.

### 4.5 DLL4 notch as a potential autocrine pathway of IL‐33 expression

One pathway that is activated in PAH is the Notch signaling pathway. In particular, Notch3‐HES signaling in PAH has been implicated in smooth muscle cell proliferation (Li et al., 2009; Smith et al., 2015). Previous reports in the literature also support Notch signaling as a known driver of IL‐33 expression and, the notch ligand, DLL4, is a strong inducer of IL‐33 transcripts in other endothelial cells, i.e. human umbilical vein‐derived endothelial cells (HUVECs), human dermal microvascular endothelial cells from juvenile foreskin (HDMECs) and human pulmonary microvascular endothelial cells (HPMECs; Sundlisaeter et al., 2012). Our data show increased cellular IL‐33 mRNA in hPAECs treated with exogenous IL‐33 and, simultaneously, stimulated by extracellular Dll4‐Notch (Figure 5). Furthermore, at the protein level, exogenous IL‐33 increases cellular IL‐33 greater than DLL4 (Figure 5). This finding suggests that a potential autocrine mechanism may be initiated in endothelial cells and, activated endothelial cells could signal smooth muscle cells. Several studies have investigated the mechanisms by which IL‐33 is released into the extracellular space but, a mechanism by which Dll4 regulates this release is incomplete (Luzina et al., 2019; Momota et al., 2020; Travers et al., 2018). An elegant study by Kakkar et al. (Chen et al., 2015; Kakkar et al., 2012) revealed dynamic trafficking of IL‐33 between the nucleus and the cytoplasm. In the cytoplasm, IL‐33 is contained in membrane‐bound vesicles and, a major signal to release these vesicles is mechanical strain. IL‐33 is highly expressed in quiescent endothelial cells and Dll4‐induces quiescence. Therefore, with increased strain, as may occur in pulmonary hypertensive vessels, IL‐33 could be released, and Dll4 could upregulate its expression as the endothelial barrier is restored. However, it should be noted that many signaling mechanisms regulate IL‐33 and, our in vitro data illustrate a critical potential mechanism (Pinto et al., 2018).

## 5 SUMMARY

Understanding the role of inflammatory processes in the development of PH is an area of increasing importance. We investigated the role of the IL‐33/ST2/MYD88 axis in the progression of the disease. We find in our mouse model that IL‐33/ST2 is essential for the endothelial cell hyperproliferative response induced by SUHX that leads to remodeling. In addition, we find that the intracellular TIR adaptor protein, MYD88, synergizes with the IL‐33/ST2 receptor complex to regulate RVP and RV contractility and remodeling. Interestingly, the expression and release of IL‐33 is dependent on SUHX. DLL4 may provide an alternative pathway for IL‐33 expression and secretion in pulmonary artery endothelial cells. Further studies are needed to determine the role of DLL4‐notch mediated IL‐33 signaling in the development of PH in the SUHX mouse model and to confirm the role of endothelial expressed IL‐33 with targeted endothelial IL‐33 gene deletion, a limitation of the present study.

## CONFLICT OF INTEREST

The authors of this study have no conflicts of interest to declare.

## AUTHOR CONTRIBUTIONS

Experimental design—ECB, CSI, TDB, MMF; data collection and analyses—CSI, AKG, TT, DTC, SCJ, ECB; preparation of manuscript—CSI, MMF, PAJ, ECB. Authors (CSI, AKG, TT, DTC, SCJ, MMF, PAJ, TDB, ECB) have read and approved the manuscript.

## Supporting information



Supplementary Material
