# Supplementary material for: IL‐33/ST2 receptor‐dependent signaling in the development of pulmonary hypertension in Sugen/hypoxia mice
Source: Physiol Rep. 2022 Feb 12;10(3):e15185. doi: 10.14814/phy2.15185 (PMC8839421; doi:10.14814/phy2.15185)
Supplement: Supplementary file 1 — Supplementary Material [file PHY2-10-e15185-s001.docx]

**Online Supplement Data**

**Supplementary Data**


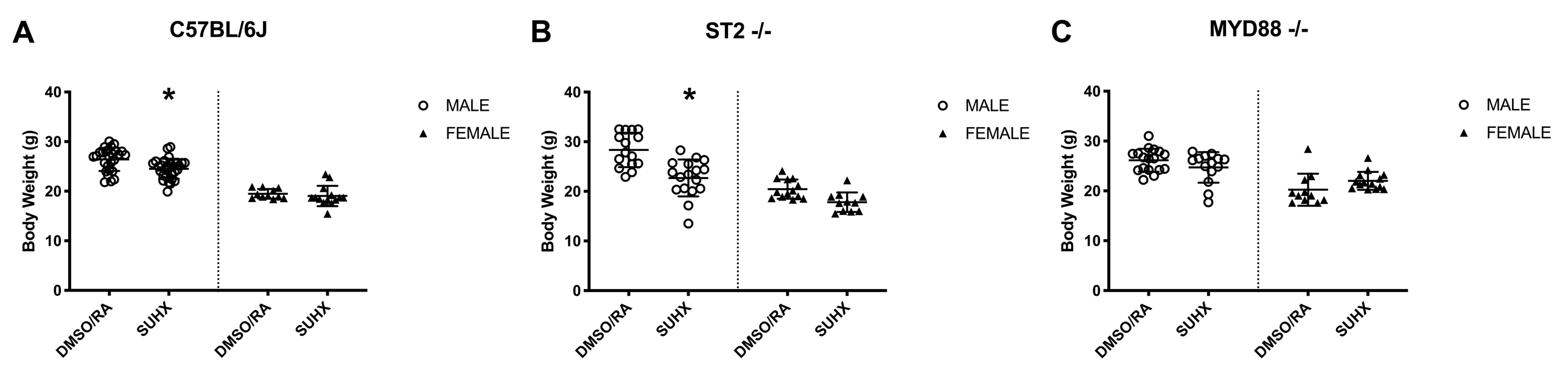


**Figure S1. Effect of SUHX conditions on body weight.** Body weight (g) measurements of male and female (A) C57BL/67, (B)ST2-/- and (C) MYD88-/- mice after 3 weeks under DMSO/RA and SUHX conditions. Values are presented as mean $\pm$ SD (n = 15-27 males, n = 11-13 females, DMSO/RA; n = 14-29 males, n = 11-14 females SUHX). * Indicates statistical significance between DMSO/RA and SUHX treatments within each sex, p< 0.05.

**SUHX dependent weight loss in male mice.** Long-term exposure to hypoxia may also be reflected in the loss of overall body mass. Therefore, body mass was recorded in C57BL/67, ST2-/- and MYD88-/- mice after 3 weeks of either DMSO/RA or SUHX treatments. The male C57BL/6J mice under SUHX conditions weighed 19.9% less than C57BL/67 under DMSO/RA (p=0.002). Male ST2-/- mice exposed to SUHX weighed 7.2% less than male ST2-/- kept in DMSO/RA (p<0.0001) (Fig. S1). Female C57BL/67 (p=0.83), ST2-/- and both sexes of MYD88-/- mice did not show a difference in body mass following between SUHX or DMSO/RA treatments (ST2-/- female p=0.08; MYD88-/- male p=0.24, female p=0.18) (Fig. S1).

**SUHX dependent weight loss in male mice**

Only the male WT and ST2-/- mice showed a decrease in body weight after SUHX treatment (Fig. S1). The sex difference of the body weight loss in WT and ST2-/- mice exposed to SUHX can be explained by the presence of estrogen (Fig. S1). Research shows that the body weight decreased significantly in SUHX exposed placebo- and estrogen-treated OVX female mice (1). The weight loss is significantly less in the estrogen treated group (1).

References

**Supplementary Methods**

**Immunohistochemistry** was done on lung sections using the following protocol: several washes in a d-Limonene-based solvent (CitroSolv, Deacon Laboratories Inc., King of Prussia, PA, USA) heated to 60°C, 3 X 5 min PBS, graded dehydration in ethanol (100%, 95%, 90%,70%, and 50% ethanol), washed with 0.1% PBS-Tween20 5 min, antigen retrieval step for 5 mins at 95°C (Dako Target Retrieval Solution Agilent, Santa Clara, CA, USA). Sections were then incubated for 1 hr at room temperatures (RT) in blocking solution [1.5% normal goat serum (NGS)/1.5% bovine serum albumin (BSA) in PBS] followed by overnight incubation with primary antibodies at 4°C: Ki-67 (Thermo Fisher Cat #50245564; 10:1000) and α-SMA-eFluor660 (eBioscience 50976080; 1:1000). Signals were detected with Alexa Fluor 546, 1:1000 (Invitrogen, Carlsbad CA, USA) 30 mins RT and mounted with Pro-long Gold with DAPI (Thermo Fisher). Digital images were acquired using Olympus FV100 Confocal with SIM Scanner and the final composites were processed using ImageJ.

**Cell Experiments**. hPAECs were cultured in Endothelial Cell Growth Supplement (EnGS) media (Lifeline Cell Technology, Frederick, MD, USA) with penicillin (50U/mL) and streptomycin (50ug/mL). All cells were from passage 4 to 5. hPAECs were seeded in 6-well plates at a density of 3.0x105 cells/cm2 cultured with 0.2% gelatin (w/v) in phosphate buffered saline (PBS) with or without rh DLL4 (1ug/mL) and treated with IL-33 protein (100ng/mL) (R&D Systems).Total RNA was extracted from hPAECs with a RNeasy mini kit (Qiagen, Valencia, CA, USA). RNA was reverse-transcribed using SuperScript Reverse Transcriptase III with Oligo(dT) primers (Life Technologies, Carlsbad, CA, USA). Transcripts were amplified with gene specific primers (Table S1.) and SYBR green master mix (Eurogentech, LIEGE Science Park, Belgium) using a quantitative PCR instrument (StepOnePlus, Life Technologies, Carlsbad, CA, USA).

Table S1: qPCR primer sequences

| Gene | Primers |
| --- | --- |
| GAPDH | Fwd 5’ - ACAGTCAGCCGCATCTTCTT - 3’  Rev 5’ - TGGAAGATGGTGATGGGATT - 3’ |
| IL-33 | Fwd 5’ - GGAGTGCTTTGCCTTTGGTA - 3’  Rev 5’ - TCATTTGAGGGGTGTTGAGA - 3’ |
| sST2 | Fwd 5’ - CTGTCTGGCCCTGAATTTGC - 3’  Rev 5’ - TGGAACCACACTCCATTCTGC - 3’ |
| ST2L | Fwd 5’ - AGGCTTTTCTCTGTTTCCAGTAATCGG - 3’  Rev 5’ - GGCCTCAATCCAGAACATTTTTAGGATGATAAC - 3’ |

Measurement of GAPDH was used as an internal control. Levels of IL-33 were measured in the cell layer using a human IL-33 ELISA kit from R&D Systems.

1. Liu A, Schreier D, Tian L, Eickhoff JC, Wang Z, Hacker TA, Chesler NC. Direct and indirect protection of right ventricular function by estrogen in an experimental model of pulmonary arterial hypertension. *Am J Physiol Heart Circ Physiol* 2014;307(3):H273-283.
